# Effect of Ethyl Pyruvate on Skeletal Muscle Metabolism in Rats Fed on a High Fat Diet

**DOI:** 10.3390/nu5072372

**Published:** 2013-07-01

**Authors:** Robert A. Olek, Wieslaw Ziolkowski, Tomasz H. Wierzba, Jan J. Kaczor

**Affiliations:** 1 Biochemistry Department, Gdansk University of Physical Education and Sport, Gorskiego 1, 80-336 Gdansk, Poland; E-Mails: wiech@awf.gda.pl (W.Z.); kaczor@awf.gda.pl (J.J.K.); 2 Physiology Department, Medical University of Gdansk, Debinki 1, 80-211 Gdansk, Poland; E-Mail: twierzba@gumed.edu.pl; 3 Department of Bioenergetics and Physiology of Exercise, Medical University of Gdansk, Debinki 1, 80-211 Gdansk, Poland

**Keywords:** mitochondrial metabolism, skeletal muscle, sulfhydryl groups, insulin, obesity, diet

## Abstract

Impaired mitochondrial capacity may be implicated in the pathology of chronic metabolic diseases. To elucidate the effect of ethyl pyruvate supplementation on skeletal muscles metabolism we examined changes in activities of mitochondrial and antioxidant enzymes, as well as sulfhydryl groups oxidation (an indirect marker of oxidative stress) during the development of obesity. After 6 weeks feeding of control or high fat diet, Wistar rats were divided into four groups: control diet, control diet and ethyl pyruvate, high fat diet, and high fat diet and ethyl pyruvate. Ethyl pyruvate was administered as 0.3% solution in drinking water, for the following 6 weeks. High fat diet feeding induced the increase of activities 3-hydroxyacylCoA dehydrogenase, citrate synthase, and fumarase. Moreover, higher catalase and superoxide dismutase activities, as well as sulfhydryl groups oxidation, were noted. Ethyl pyruvate supplementation did not affect the mitochondrial enzymes’ activities, but induced superoxide dismutase activity and sulfhydryl groups oxidation. All of the changes were observed in soleus muscle, but not in extensor digitorum longus muscle. Additionally, positive correlations between fasting blood insulin concentration and activities of catalase (*p* = 0.04), and superoxide dismutase (*p* = 0.01) in soleus muscle were noticed. Prolonged ethyl pyruvate consumption elevated insulin concentration, which may cause modifications in oxidative type skeletal muscles.

## 1. Introduction

Mitochondria play a key role in the energy metabolism of skeletal muscles. Impaired mitochondrial oxidative capacity in skeletal muscles may be implicated in the pathology of chronic metabolic disease characterized by insulin resistance such as obesity, type 2 diabetes mellitus, and aging [1,2]. However, it is not clear whether mitochondrial dysfunction is a cause or consequence of metabolic disorders. It has been demonstrated that exposure to high fat diet (HFD) reduces mitochondrial oxidative capacity [3,4,5]. The 6 weeks HFD treatment causes significant decrease in oxidative phosphorylation activity of rat *soleus* muscles [4,6]. Moreover, it has been reported that the mitochondrial dysfunction in skeletal muscle from HFD fed rodents is associated with alterations in oxidative stress markers [4,7,8,9]. Since in the presence of saturated fatty acids mitochondria are potential source of reactive oxygen species (ROS) [10,11], increased oxidative stress in skeletal muscle may disrupt mitochondrial enzymes thereby resulting in decreased oxidative metabolism [12,13,14].

Muscle oxidative capacity depends mainly on mitochondrial biogenesis and the mitochondrial enzyme activity. A number of modulators have been involved in the regulation of muscle mitochondrial biogenesis and oxidative phosphorylation activity [15]. It has been shown that prolonged pyruvate treatment of C2C12 myotubes upregulated mitochondrial proteins and mRNAs for those proteins [16]. Moreover, pyruvate is an energetic substrate [17], which may alter the metabolism of obese rats [18]. Furthermore, it can act as an antioxidant [19]. By a nonenzymatic reaction it reduces hydrogen peroxide to water [20] and scavenges hydroxyl radical [21]. The effectiveness of ethyl pyruvate (EtP) has been proven in various stress conditions [22,23,24,25,26,27].

Therefore, we assumed that 6 weeks of HFD would induce metabolic dysfunction, and the inclusion of EtP supplementation may have some beneficial effect on skeletal muscle mitochondrial and antioxidant enzymes activities, as well as sulfhydryl groups (SH) oxidation—an indirect marker of oxidative stress. *Soleus* (SOL) and *extensor digitorum longus* (EDL) muscles were used to evaluate if the prospective changes are fiber-type specific.

## 2. Experimental Section

### 2.1. Animals and Diets

Thirty-two male Wistar rats at the age of 7 weeks were obtained from the Center of Experimental Medicine at the Medical University of Bialystok (Poland). After a 1-week familiarization period, the rats were divided randomly into 2 groups. The control group (*n* = 16; 201 ± 4 g) was fed a standard maintenance diet contained 12.8 MJ/kg metabolizable energy, with 9% of its energy from fat, 33% from protein, and 58% from carbohydrates; including 6.6% of sucrose (V1534-000 ssniff R/M-H, ssniff Spezialdiäten GmbH, Soest, Germany). The diet group (*n* = 16; 201 ± 3 g) was fed a HFD composed as previously described [4]. HFD containing 19.5 MJ/kg metabolizable energy, with 45% of its energy from fat, 17% from protein, and 38% from carbohydrates (ssniff Spezialdiäten GmbH, Soest, Germany). The HFD derived its fat from lard (31%), peanut oil (7%), and canola seed oil (7%); carbohydrates from cornstarch (26%) and sucrose (12%). Animals had free access to food and water and were kept at room temperature with a light-dark cycle of 12 h. After 6 weeks, both groups were subdivided into 4 groups: control diet (CC; *n* = 8), control diet and EtP (CP; *n* = 8), HFD (DC; *n* = 8), HFD and EtP (DP; *n* = 8). EtP was administered as 0.3% EtP solution in drinking water for the following 6 weeks [28]. At the end of 12th week, the rats were sacrificed. The excised SOL and EDL muscles were immediately frozen in liquid nitrogen. The blood was centrifuged at 2000 *g* for 10 min at 4 °C. Separated plasma and red blood cells, as well as skeletal muscle samples were stored at −70 °C for later analyses. All procedures were approved by the Local Animal Ethics Committee and performed in accordance with guidelines for animal care.

### 2.2. Enzymes Activities and Sulfhydryl Groups Oxidation

Prior to the chemical assays, muscles were minced and homogenized in an ice-cold buffer that contained 50 mM potassium phosphate, 1 mM ethylenediaminetetraacetic acid (EDTA), 1 mM threo-1,4-dimercapto-2,3-butanediol (DTT) at pH 7.4. The homogenates were then centrifuged at 600 *g* at 4 °C for 10 min to rid them of cellular debris. Enzyme activities and SH group concentration were determined in the obtained supernatant using a Super Aquarius CE9200 spectrophotometer (Cecil Instruments Ltd., Cambridge, UK).

3-hydroxyacylCoA dehydrogenase (HADH) activity was determined in a buffer containing 100 mM potassium phosphate and 0.05% Triton at pH 7.4. After addition of supernatant and 0.1 mM NADH the cuvette was incubated for 3 min at 30 °C. The reaction was started by the acetoacetyl-CoA (0.1 mM final concentration) and the change in absorbance at 340 nm was followed in time. Enzyme activity was calculated using molar absorption coefficient of NADH 6220 M^−1^ cm^−1^.

Citrate synthase (CS) activity was measured by the rate of SH production as CoASH using the thiol reagent 5,5′-dithiobis (2-nitrobenzoic acid) (DTNB). DTNB reacts spontaneously with SH to produce a free thionitrobenzoate anion, which has an absorption maximum at 412 nm. The reagent cocktail contained 50 mM potassium phosphate, 0.1 mM DTNB, and 0.1 mM acetylCoA. The reaction was started by the addition of 0.1 mM (final concentration) oxaloacetic acid (adjusted to pH 7.4).

Fumarase (Fum) activity was assayed in the mixture containing 30 mM potassium phosphate, 0.1 mM EDTA at pH 7.4. The reaction was started by the addition of 5 mM l-malate. The increase in absorbance at 240 nm was monitored and the enzyme activity was calculated using a molar absorption coefficient 2440 M^−1^ cm^−1^.

Catalase (CAT) activity was measured in the mixture containing 50 mM potassium phosphate, 5 mM EDTA, 0.01% Triton at pH 7.4. The reaction was started by the addition of hydrogen peroxide (H_2_O_2_). The kinetic of H_2_O_2_ decomposition was followed in time at 240 nm, and CAT activity was calculated using a molar absorption coefficient 43.6 M^−1^ cm^−1^.

Superoxide dismutase (SOD) activity was assayed using standard test kits (Randox Laboratories Ltd., Crumlin, UK). This method employs xanthine and xanthine oxidase to generate superoxide radicals which react with 2-(4-iodophenyl)-3-(4-nitrophenol)-5-phenyltetrazolium chloride (INT) to form a red formazan dye. The SOD activity is then measured by the degree of inhibition of this reaction. One unit of SOD is that which causes a 50% inhibition of the rate of reduction of INT under the conditions of the assay.

The SH group concentration was determined according to Ellman’s method [29]. Briefly, samples were incubated with 0.1 mM DTNB at room temperature for 60 min. Absorbance was determined at 412 nm. Protein content was evaluated by the Lowry *et al.* method [30].

### 2.3. Plasma Biochemical Analyses

Plasma insulin was determined by enzyme-linked immunosorbent assay kit from EMD Millipore Corp. (Cat. #EZRMI-13K). Glucose and glycosylated hemoglobin (HbA1c) were measured using commercial assay kits (Randox Laboratories Ltd., Crumlin, UK).

### 2.4. Chemicals

All reagents were obtained from Sigma-Aldrich, unless otherwise stated.

### 2.5. Statistical Analyses

All results are expressed as the means ± standard error (SE). Comparisons among groups were conducted by two-way analyses of variance (ANOVA) with Fisher post-hoc test using STATISTICA 9.0 (Statsoft Inc., Tulsa, OK, USA) software. Pearson’s correlation coefficient was assessed to estimate the degree of association between two numerical variables. The cut-off for significance was set at *p* < 0.05.

## 3. Results

Twelve weeks of HFD treatment induced a significant increase in rat body mass (main effect *p* < 0.005). However, six weeks of EtP supplementation did not influence the weight either in CP or in DP groups. The increase in rat mass was 222 ± 12; 216 ± 8; 252 ± 7; 251 ± 10 g in CC, CP, DC and DP groups, respectively.

HFD feeding induced the increase of mitochondrial enzymes activities in SOL, but not in EDL (Table 1). EtP supplementation for the last 6 weeks did not affect the oxidative metabolism either in SOL or in EDL (Table 1).

**Figure 1:**
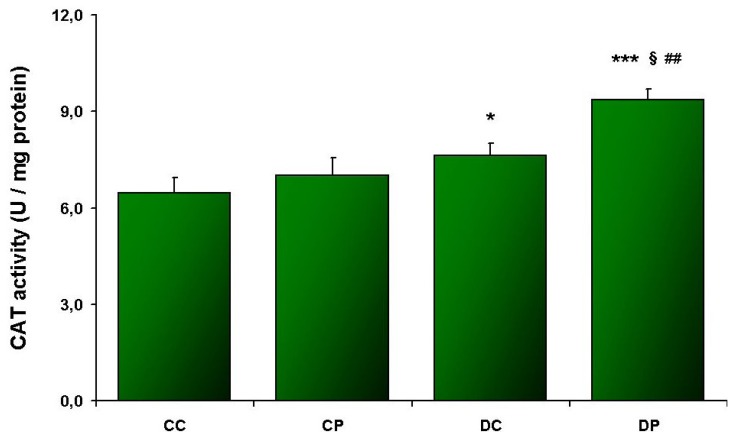
Catalase activity in SOL muscle; control diet (CC), control diet and EtP (CP), HFD (DC), HFD and EtP (DP). Values are means ± SE. * *p* < 0.05 as compared to CC; *** *p* < 0.005 as compared to CC; § *p* < 0.005 as compared to CP; ## *p* < 0.01 as compared to DC.

**Figure 2:**
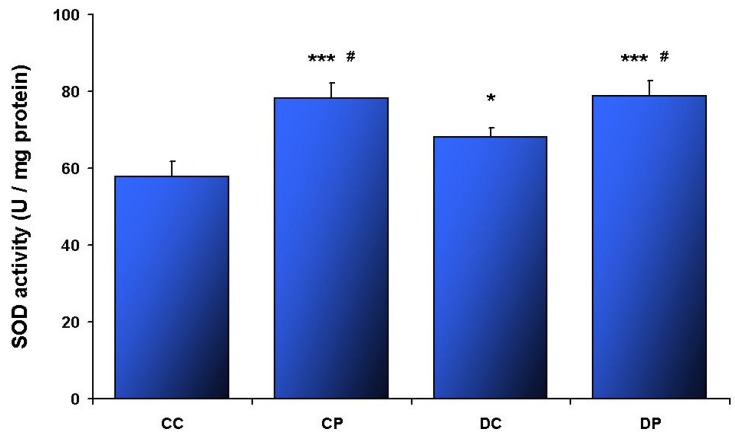
Superoxide dismutase activity in SOL muscle; control diet (CC), control diet and EtP (CP), HFD (DC), HFD and EtP (DP). Values are means ± SE. * *p* < 0.05 as compared to CC; *** *p* < 0.005 as compared to CC; # *p* < 0.05 as compared to DC.

**Figure 3:**
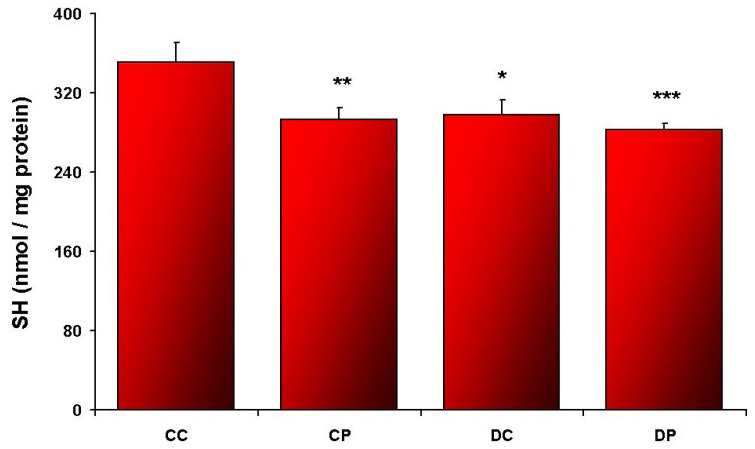
Sulfhydryl groups concentration in SOL muscle; control diet (CC), control diet and EtP (CP), HFD (DC), HFD and EtP (DP). Values are means ± SE. *p < 0.05 as compared to CC; ** p < 0.01 as compared to CC; *** p < 0.005 as compared to CC.

Elevated CAT (Figure 1) and SOD (Figure 2) activities, as well as accelerated SH oxidation (Figure 3) were noted in SOL muscle obtained from HFD rats. CAT and SOD activities were increased in DP compared to DC (*p* < 0.01; *p* < 0.05, respectively). Additionally SOD activity in CP was higher compared to CC (*p* < 0.005), and DC (*p* < 0.05). Such changes were not observed in EDL muscle (Table 2).

**Table 1:** Activities of oxidative metabolism enzymes in *soleus* (SOL) and *extensor digitorum longus* (EDL) muscles after 12 weeks of treatment (means ± SE).

	Groups			
	CC	CP	DC	DP
*SOL*				
HADH (mU/mg protein) *	133 ± 9	134 ± 6	180 ± 12	184 ± 5
CS (mU/mg protein) **	151 ± 16	150 ± 4	179 ± 14	168 ± 8
Fum (mU/mg protein) **	150 ± 12	136 ± 4	169 ± 11	155 ± 10
*EDL*				
HADH (mU/mg protein)	61 ± 5	62 ± 5	69 ± 9	63 ± 6
CS (mU/mg protein)	163 ± 14	151 ± 10	158 ± 18	140 ± 8
Fum (mU/mg protein)	124 ± 7	118 ± 8	119 ± 9	108 ± 8

* *p* < 0.001 main effect of the diet; ** *p* < 0.05 main effect of the diet.

**Table 2:** Antioxidant enzymes activities and sulfhydryl groups in EDL muscle after 12 weeks of treatment (means ± SE).

	Groups			
	CC	CP	DC	DP
*EDL*				
CAT (U/mg protein)	1.86 ± 0.07	1.54 ± 0.13	1.90 ± 0.20	1.87 ± 0.15
SOD (U/mg protein)	50.6 ± 2.0	51.0 ± 3.3	51.1 ± 7.6	45.1 ± 2.4
SH (nmol/mg protein)	272 ± 5	262 ± 7	275 ± 11	257 ± 2

Plasma insulin concentration after 12 weeks of HFD was higher than in control rats. Moreover, EtP supplementation triggered the elevation of insulin in both groups CP and DP. The concentration of glucose and HbA1c did not change significantly in either group (Table 3).

**Table 3:** Plasma insulin, glucose and HbA1c in rats after 12 weeks of treatment (means ± SE).

	Groups			
	CC	CP	DC	DP
Insulin (ng/mL)	0.49 ± 0.14	1.48 ± 0.28 *	1.19 ± 0.27 **	1.52 ± 0.19 ***
Glucose (mg/dL)	92.6 ± 1.8	94.5 ± 2.2	94.1 ± 2.8	96.3 ± 2.4
HbA1c (%)	4.83 ± 0.13	4.73 ± 0.10	4.70 ± 0.05	4.71 ± 0.05

* *p* < 0.01 as compared to CC; ** *p* < 0.05 as compared to CC; *** *p* < 0.005 as compared to CC.

There was a weak but significant positive correlation between plasma insulin concentration and CAT (*r*^2^ = 0.16; *p* = 0.04), and SOD (*r*^2^ = 0.23; *p* = 0.01) in SOL muscle.

## 4. Discussion

In the current study, 12 weeks of HFD rats treatment caused an increase in mitochondrial and antioxidant enzymes activities only in SOL muscle, which was associated with SH oxidation. This effect was not abolished by EtP supplementation. Moreover, EtP intake alone for 6 weeks elevated SOD activity and SH oxidation, although no modification in mitochondrial metabolism was revealed. This observation does not agree with our hypothesis that EtP supplementation would ameliorate impaired by HFD mitochondrial metabolism.

Over recent years, a number of studies have provided data to indicate that abnormalities in skeletal muscle metabolism are implicated in the development of insulin resistance. HFD treatment in animal models induced obesity and type 2 diabetes, but the evidence on mitochondrial function is contradictory. In the present study, we used a classical experimental 40%–50% HFD [3,4,5,31,32,33,34]. However, the effect of this type of diet on muscle oxidative metabolism is inconsistent. It has been shown that muscle oxidative capacity is decreased [3,4,5], or improved [31,32,33,34] after HFD. The reported discrepancies might result from different composition of the diets [5]. HFD used in our study was composed as described by Chanseaume *et al.* [4], nevertheless our data were opposite to the previously presented [4]. It has also been speculated that the age at which high-fat feeding starts is an important factor, since very young rats (25 days old) fed diets enriched with fat exhibit decreased energetic efficiency [35]. Similarly, initiation of HFD treatment in 3-month old rats impairs mitochondrial function [3,4,5]. We have started experimental protocol with 2-month old rats, but our data showed an increased oxidative capacity after HFD, which is comparable to other groups [31,32,33]. It seems that the most likely reason for the various effect of HFD on muscle mitochondria function could be due to the different susceptibility for obesity of treated animals [36]. In the studies where unfavorable effect of HFD were observed, the mass of 3-month old male Wistar rats was as high as 400–440 g at the beginning of the experimental protocol [3,5,6]. These rats are called “potentially prone to developing obesity” [3]. In the study reporting an increase of mitochondrial content after long-term HFD, the treatment was initiated at the age of 14 weeks, when the weight of the male Wistar rats was 366 ± 16 g [32]. Consistently, in our study the control rats at the age of 14 weeks were 365 ± 11 g [28], Modification in muscle metabolism may also be influenced by the duration of HFD. The chronological changes in the muscles of rats fed by HFD has been described in the study by Chanseaume *et al.* [6]. Mitochondrial oxidative capacity increases after 2 weeks, and then drops after the following 4 weeks of HFD treatment in the rats predisposed to obesity [6]. Similar changes may develop for longer period of time in the rats not predisposed to obesity. Increased muscle mitochondrial metabolism was observed both in our study after 12 weeks of the HFD as well as in the van den Broek *et al.* study after 25 weeks of HFD [32].

The response to HFD may also depend on muscle fiber type. In the present study, HFD induced changes in SOL, but not in EDL. Increased mitochondrial enzymes activities in SOL were associated with elevation of SOD and CAT activities, as well as SH oxidation. Similarly, in the recent study, it has been shown that only 5 days HFD treatment alters cellular redox state in SOL, not in EDL [37]. The authors suggested that glycolytic muscle is less susceptible to HFD induced alterations, due to reduced expression of fatty acid transporters and rates of fatty acids transport across both the sarcolemmal and mitochondrial membranes [38,39], and therefore lower ROS formation caused by long chain fatty acids [40].

Mitochondrial protein content and oxidative capacity of the muscles are controlled by the number of transcription factors [15]. HFD induces mitochondrial biogenesis by increasing of peroxisome proliferator-activated receptor gamma coactivator-1 alpha (PGC-1α), and beta (PGC-1β) [31,34]. Since elevation in muscle mitochondrial content may be associated with ROS overproduction [11], defensive mechanisms must also be improved. It has been reported that PGC-1β overexpression in rat muscle is sufficient to enhance mitochondrial enzymes expression, but also to ameliorate antioxidant defense [34]. Wilson *et al*. [16] found that supraphysiological levels of sodium pyruvate induced mitochondrial biogenesis in myoblast cells, but this effect was independent of PGC-1α and PGC-1β mRNA expression. Since pyruvate in the aqueous solutions transforms into parapyruvate—an inhibitor of the tricarboxylic acid (TCA) cycle [41], we have used EtP which is a more stable compound. However, our results demonstrated no relevant modification in mitochondrial enzyme-activity. Moreover, we observed accelerated SH oxidation and increased antioxidant enzymes activity in EtP treated groups. Despite the well known scavenging properties of EtP [42], recent studies have shown accelerated mitochondrial ROS production in the presence of EtP [43]. The authors suggested that EtP may act as an ROS inducer through initiation of the TCA cycle [43]. In the present study activities of the mitochondrial enzymes were not altered by the EtP treatment, which may indicate that changes in oxidative type skeletal muscle may be accelerated by non-mitochondrial generating system(s). Recently, it has been found that insulin and glucose infusion selectively enhance ROS production in muscle through xanthine oxidase [11]. This effect is acute and not damaging [11]. We found a positive correlation between insulin concentration with SOD and CAT activities in SOL, which may imply the influence of xanthine oxidase system in oxidative skeletal muscle cell alterations.

Six weeks of treatment with 0.3% EtP in drinking water induced increase in serum insulin concentration in our study. Similar results were reported in broiler chickens supplemented with creatine pyruvate [44]. However, the birds were fed with a diet enriched with 5% and 10% of the compound for a period of 3 weeks; in the group supplemented with 1% creatine pyruvate this effect was not observed [44]. Higher insulin concentration suggests the increase of insulin resistance. In contrast, a 6% calcium pyruvate or a pyruvylglycine in obese Zucker rats decreased insulin concentration and improved insulin sensitivity [18]. Different effects may result from various rat models. Ivy *et al*. [18] investigated hyperinsulinemic animals, whereas in the present study insulin concentration was much lower even after 12 weeks of HFD treatment. Moreover, the forms of pyruvate used as a supplement may play a pivotal role. It has been shown that properties varies between pyruvate esters and salts with the higher effectiveness of EtP than its salt [22,23,24,25,26,27]. Furthermore, anti-inflammatory properties of EtP have been related to a reduction of c-Jun NH_2_-terminal kinase phosphorylation [45,46], which is an important signaling protein involved in the skeletal muscle insulin resistance pathway [47]. Thus, EtP treatment may influence insulin sensitivity; however, the fact that we did not measure insulin resistance is the main limitation of the current study.

## 5. Conclusions

EtP is used as a food additive (JECFA No. 938) [48], therefore the effect of its consumption may be of practical importance. In the present study, HFD elevated skeletal muscle mitochondrial enzymes activities, but EtP supplementation was without effect. However, EtP induced modifications in SOL muscle, which were related to an increase of plasma insulin concentration. Future studies should focus on the effect of EtP supplementation on glucose and insulin tolerance tests and analysis of pancreatic beta cells.
